# Deprescribing Leads to Improved Energy Intake among Hospitalized Older Sarcopenic Adults with Polypharmacy after Stroke

**DOI:** 10.3390/nu14030443

**Published:** 2022-01-19

**Authors:** Ayaka Matsumoto, Yoshihiro Yoshimura, Hidetaka Wakabayashi, Eiji Kose, Fumihiko Nagano, Takahiro Bise, Yoshifumi Kido, Sayuri Shimazu, Ai Shiraishi

**Affiliations:** 1 Department of Pharmacy, Kumamoto Rehabilitation Hospital, Kumamoto 869-1106, Japan; ayk1224mtmt@gmail.com; 2 Center for Sarcopenia and Malnutrition Research, Kumamoto Rehabilitation Hospital, Kumamoto 869-1106, Japan; 3 Department of Rehabilitation Medicine, Tokyo Women’s Medical University Hospital, Tokyo 162-8666, Japan; noventurenoglory@gmail.com; 4 Department of Pharmacy, Teikyo University School of Medicine University Hospital, Tokyo 173-8606, Japan; kose.eiji@med.teikyo-u.ac.jp; 5 Department of Rehabilitation, Kumamoto Rehabilitation Hospital, Kumamoto 869-1106, Japan; f-nagano@kumareha.jp (F.N.); asian.dub.foundation00@gmail.com (T.B.); kidonii921@yahoo.co.jp (Y.K.); 6 Department of Nutritional Management, Kumamoto Rehabilitation Hospital, Kumamoto 869-1106, Japan; shimazu@kumareha.jp; 7 Department of Dental Office, Kumamoto Rehabilitation Hospital, Kumamoto 869-1106, Japan; ai.shiraishi0913@gmail.com

**Keywords:** polypharmacy, deprescribing, rehabilitation pharmacotherapy, sarcopenia, rehabilitation nutrition

## Abstract

Evidence is scarce regarding the polypharmacy in patients with sarcopenia. The aim of this study was to investigate the effect of deprescribing for polypharmacy on the improvement of nutritional intake and sarcopenia in older patients with sarcopenia. A retrospective cohort study was conducted with hospitalized older patients with sarcopenia undergoing rehabilitation after stroke. Study outcomes included energy intake, protein intake, handgrip strength (HG) and skeletal muscle mass index (SMI) at hospital discharge. To consider the effects of deprescribing for polypharmacy, we used multivariate analyses to examine whether the change in the number of medications during hospitalization was associated with outcomes. Of 361 patients after enrollment, 91 (mean age 81.0 years, 48.4% male) presented with sarcopenia and polypharmacy and were eligible for analysis. The change in the number of medications was independently associated with energy intake (β = −0.237, *p* = 0.009) and protein intake (β = −0.242, *p* = 0.047) at discharge, and was not statistically significantly associated with HG (β = −0.018, *p* = 0.768) and SMI (β = 0.083, *p* = 0.265) at discharge, respectively. Deprescribing was associated with improved nutritional intake in older sarcopenic patients with polypharmacy undergoing stroke rehabilitation.

## 1. Introduction

Nutritional management is important for older adults with sarcopenia. Sarcopenia is a disease mainly caused by aging, disease, low activity, and malnutrition, and diagnosed by decreased skeletal muscle mass, muscle strength, and physical function [1]. Sarcopenia and malnutrition commonly occur in older adults [2,3] and are associated with adverse outcomes such as decline in activities of daily living (ADL), falls, fractures, dysphagia, and death [4,5]. Moreover, sarcopenia, malnutrition, weight loss, and decreased nutritional intake are independent factors that negatively affect functional recovery in hospitalized older patients [6,7,8]. Therefore, early diagnosis and appropriate treatment of sarcopenia are necessary. Exercise and nutritional therapy are the mainstays of treatment for sarcopenia [9,10]. For nutritional therapy, current guidelines recommend high protein intake and supplementation with essential amino acids, with little evidence of their benefits [10,11]. In patients undergoing rehabilitation, the efficacy of branched-chain amino acids and leucine in improving physical function and sarcopenia has been reported [12,13]. Furthermore, increasing body weight and muscle mass through adequate nutrition promote improvements in ADLs in hospitalized patients [14,15,16]. It is, therefore, clinically important to improve sarcopenia and malnutrition in older patients with aggressive nutritional management to maximize favorable outcomes.

Polypharmacy is a common health concern among older adults and is associated with malnutrition and reduced physical function. Prescribing a numerous number of drugs can lead to medication-related problems such as inappropriate medication use, duplication of therapy, adverse drug effects, drug interactions, poor adherence, unnecessary medication use, and strain on medical resources. Old age, multimorbidity, poor physical function, and low cognitive levels are associated with polypharmacy [17,18]. Recently, polypharmacy has been reported to be related to sarcopenia [19], which is one of the major risk factors for frailty [20]. Furthermore, excessive polypharmacy (>10 drugs) is associated with an increased risk of malnutrition after 3 years in older adults aged >75 years [21]. Therefore, prevention and modification of polypharmacy may lead to improvement in sarcopenia, malnutrition, and physical function in older adults.

However, there is a lack of evidence on the association between modifying polypharmacy and improvement of nutritional status and sarcopenia, which are associated with negative rehabilitation outcomes [5,20]. Clarifying this relationship would help highlight the importance of drug therapy or pharmacotherapy in rehabilitation medicine.

Therefore, we aimed to investigate the effect of deprescribing to reduce polypharmacy on the improvement of nutritional intake and sarcopenia in older patients with sarcopenia undergoing rehabilitation after stroke.

## 2. Materials and Methods

### 2.1. Participants and Setting

We conducted a retrospective cohort study of patients admitted to a post-acute care hospital with 135 beds in convalescent rehabilitation wards. The research was conducted between January 2015 and December 2020. In this study, all newly admitted stroke patients to the rehabilitation wards were enrolled. Among them, patients aged 65 years or older who were diagnosed with sarcopenia at the time of admission and who were using six or more drugs were included in the study. Exclusion criteria included consciousness impairment of more than three digits on the Japan Commerce Scale [22], refusal to participate, missing data, altered hydration status, obvious edema, pacemaker implantation that might interfere with bioelectrical impedance analysis (BIA), and transferring to another hospital or ward during hospitalization, for acute care purposes or other reasons. The study period (observation period) for each patient was the period of hospitalization (admission date to discharge date). In this study, rehabilitation was conducted from the first day of admission to the wards until the day of discharge, and the duration of hospitalization (observation period) was the same as the duration of rehabilitation.

### 2.2. Data Collection

We recorded basic information such as age, sex, type of stroke, history of stroke, body mass index, and the number of days between stroke onset and admission to the hospital ward. A validated nutritional screening tool, the geriatric nutritional risk index (GNRI) [23], was used to assess nutritional status. Swallowing status or dysphagia was assessed by trained nurses using the Food Intake Level Scale (FILS) [24], a validated 10-point observer rating scale to evaluate swallowing status. The severity of comorbidities using the Charlson Comorbidity Index (CCI) [25] and ADL before stroke using the modified Rankin Scale (mRS) [26] were assessed by physicians.

During the first 72 h of hospitalization, skeletal muscle mass by bioimpedance analysis (BIA), handgrip strength (HG), Functional Independence Measure (FIM) scores for physical function (FIM-motor) and cognitive function, and sum of physical and cognitive function (FIM-total) [27] were measured. We measured the HG of the non-dominant hand (non-paralyzed hand in the case of hemiplegia) using a Smedley hand dynamometer (TTM, Tokyo, Japan) and recorded the highest value among the three measurements. We measured BIA using a standard protocol with the InBody S10 (InBody, Tokyo, Japan), a validated BIA instrument that has been reported to minimally affect muscle mass estimation by fluid overload [28].

### 2.3. Polypharmacy

Information on medications was obtained through medical record review. Medication information at the time of admission was routinely recorded by pharmacists, and the information at the time of discharge was recorded according to the discharge prescription issued by attending physicians. Among all prescriptions, only regularly prescribed oral medications were included in the study. Medications for transient acute illnesses (antibiotics for infections such as urinary tract infections and pneumonia, etc.), patch medications, eye drops, nasal drops, drugs for use as needed, and over-the-counter medications were excluded from the analysis. Medication at the time of hospitalization is likely to be affected by acute care, while medication at the time of discharge is likely to be affected by rehabilitative care. In this study, polypharmacy was defined as the use of six or more medications, because increased risk of adverse drug events has been reported in hospitalized older adults using six or more medications [29].

Potentially inappropriate medications (PIMs) were defined according to the American Geriatrics Society’s 2019 Beers Criteria [30] and may occur in many older adults. These criteria are used widely in the field of geriatric medicine and are among the most frequently used tools to facilitate screening for PIM.

The number of changes in medications during hospitalization was calculated by subtracting the number of medications at the time of hospitalization from the number of medications at the time of discharge.

### 2.4. Sarcopenia Definition

Sarcopenia was diagnosed when both low skeletal muscle mass index (SMI) and low HG were present, based on the Asian Working Group for Sarcopenia 2019 criteria and their cut-off values [4]. The cut-off values for SMI used for diagnosis of sarcopenia were <7 kg/m^2^ for men and <5.7 kg/m^2^ for women, and the cut-off values for HG were <28 kg for men and <18 kg for women, respectively.

### 2.5. Energy and Protein Intakes

Energy and protein intakes were estimated by trained nurses and dietitians who determined visually the ratio of actual intake to the amount provided to the patients. During the first three days of hospitalization, we recorded the intake of three meals each for breakfast, lunch, and dinner (nine meals in total), and the average of each value divided by three was used as the daily intake [31]. If the nutritional intake method of the patients was enteral nutrition (EN) or parenteral nutrition (PN), the amount of energy and protein during the first 3 days of hospitalization were recorded, and each value was divided by 3 to obtain the daily intake. If the patient’s nutritional intake was combined with oral intake and EN or PN, the respective energy and protein intakes (doses) were added. Furthermore, nutritional intake was determined by dividing each intake by the actual body weight at the time of admission. The nutritional intake of the patients was recorded both on admission and at discharge.

### 2.6. Outcomes

The main outcome was energy intake at the time of discharge. Other outcomes included protein intake, and HG and SMI values at hospital discharge.

### 2.7. Sample Size Calculation

We calculated the sample size using data from our previous study [32], the results of which showed a standard deviation of 9.2 for the patients’ energy intake (kcal/kg/day). We hypothesized that patients with decreased medication use during hospitalization would have a 10% increase in energy intake at discharge compared to those without decreased medication use. In that case, in order to reject the null hypothesis with a power of 0.8 and an alpha error of 0.05, a sample size of at least 38 people in each group would be required to support the validity of the results.

### 2.8. Statistical Analysis

Data were presented as mean (standard deviation; SD) for parametric data, median and 25–75% (interquartile range (IQR)) for nonparametric data, and numerical values (%) for categorical data. A *p* < 0.05 was set for statistical significance. Bivariable analysis was performed based on the change in the number of medications used during hospitalization and divided into two groups: one in which the number of medications decreased (deprescribing group) and one in which the number of medications increased or did not change (non-deprescribing group). Comparisons between groups were performed using *t*-test, Mann–Whitney U test, and chi-square test, depending on the type of variable data.

Multiple regression analysis was used to examine whether changes in the number of medications used were independently associated with energy intake, protein intake, HG, and SMI at hospital discharge. The baseline value (value at admission) for each outcome was included as an adjustment factor as a potential confounder for each outcome. Further, the covariates selected for adjusting for bias involved age, gender (male), FIM-total, and GNRI score at admission, all of which were assumed to be associated with nutrition intake. To minimize bias, the common confounders were adjusted for through a series of multivariate analyses. Multicollinearity was evaluated using the variance inflation coefficient, where <3 indicates no multicollinearity. All the analyses were performed using IBM SPSS version 21 (IBM, Armonk, NY, USA).

### 2.9. Ethics

The Institutional Review Board of the hospital where the study was conducted approved the study (approval number: 178-211111). Participants were able to withdraw from the study at any time through an opt-out procedure. Due to the constraints imposed by the retrospective study design, written informed consent could not be obtained. We conducted this study in accordance with the Declaration of Helsinki and the Ethical Guidelines for Medical and Health Research Involving Human Subjects.

## 3. Results

**Figure 1:**
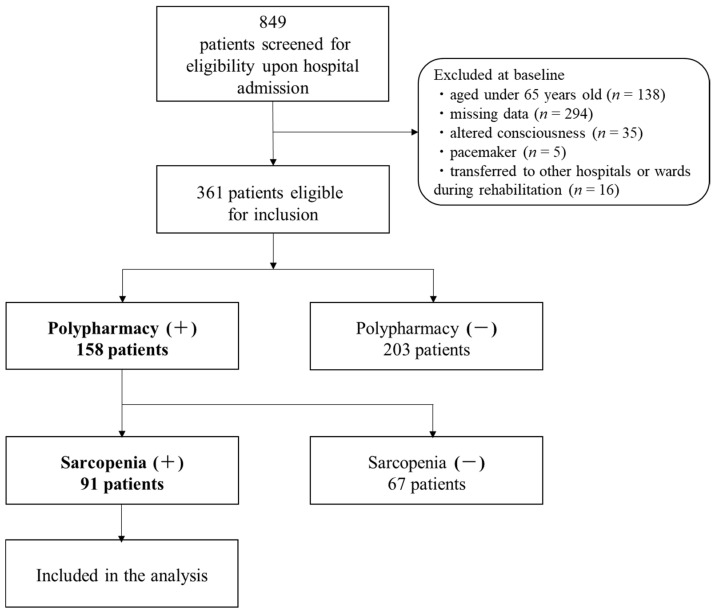
Flowchart of participant screening, inclusion criteria, and follow up.

A total of 849 stroke patients were hospitalized during the study period. Of these, patients with altered consciousness (*n =* 35), aged < 65 years (*n =* 138), pacemaker implantation (*n =* 5), missing data (*n =* 294), who were transferred to another hospital for the purpose of acute disease treatment during the study period (*n =* 16), and those using <6 medications (*n =* 203) were excluded. Then, 158 patients were screened, 91 of whom had sarcopenia at admission and were included in the final analysis (Figure 1). In this study, sarcopenia was diagnosed in 57.6% of older patients with polypharmacy after stroke.

**Figure 2:**
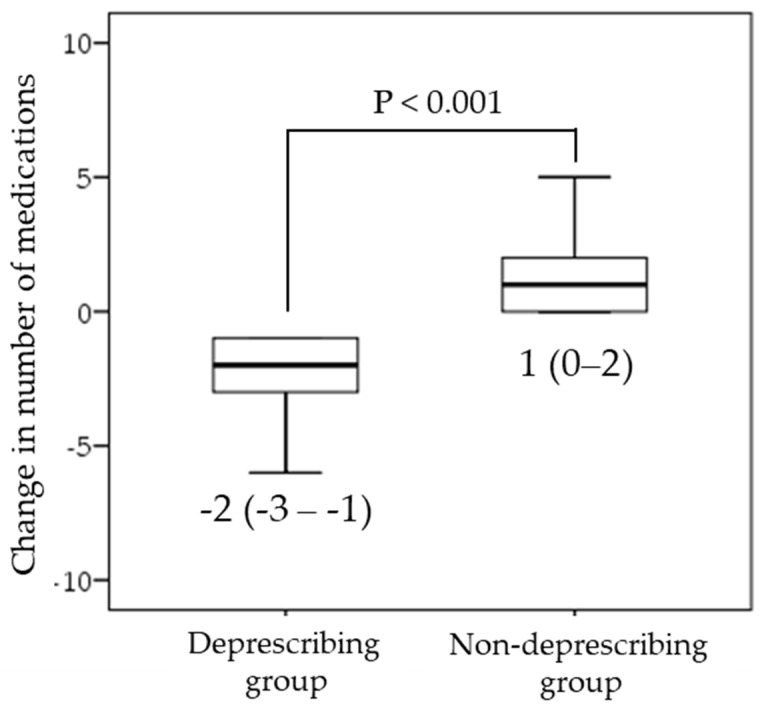
Comparison of the change in the number of medications during hospitalization between the deprescribing group and the non-deprescribing group.

Table 1 summarizes the baseline characteristics of the enrolled subjects. The mean age of the subjects was 81.0 (7.5) years, and 48.4% were male. The median number of medications prescribed at the time of admission was 8 (6–9). Medications commonly prescribed at the time of admission were antithrombotics (79%), antihypertensive agents (74%), proton pump inhibitors (67%), statins (37%), and diuretics (32%). Comparing the number of drugs at admission and discharge, there was a median decrease of two drugs in the deprescribing group and a median increase of one drug in the non-deprescribing group (Figure 2). The median GNRI score was 91 (84–99), suggesting that many of the patients were malnourished or at risk of malnutrition. The median HG values in men and women were 17.5 (10.2–22.0) kg and 9.2 (4.0–13.3) kg, respectively. The median SMI values in men and women were 6.1 (5.7–6.5) kg/m^2^ and 4.8 (4.0–5.1) kg/m^2^, respectively. The median FIM-motor score was 22 (14–51), indicating that many of the patients had physical dependence at baseline. There was no significant difference in CCI between the two groups.

Table 2 shows the comparison of the outcomes between deprescribing and non-deprescribing groups. There were no significant between-group differences in energy intake, protein intake, HG, and SMI at discharge.

Table 3 shows the results of multivariate linear regression analyses of outcomes. No multicollinearity among variables was observed. The results showed that the change in the number of medications was independently associated with energy intake (β = −0.237, *p =* 0.009) and protein intake (β = −0.242, *p =* 0.047) at discharge. There was no significant association between the change in the number of medications with HG (β = −0.018, *p =* 0.768) or SMI (β = 0.083, *p =* 0.265).

## 4. Discussion

This study examined the effects of deprescription in reducing polypharmacy on nutritional intake and sarcopenia in older patients with sarcopenia undergoing rehabilitation after stroke. We highlighted two major findings: (1) deprescribing was associated with improved nutritional intake; (2) deprescribing was not statistically significantly associated with gains in muscle strength or muscle mass in these patients.

In this study, deprescribing was associated with improved nutritional intake. Reduced number of medications during hospitalization was positively associated with an increase in energy and protein intake in stroke patients with sarcopenia. Although the association between polypharmacy and malnutrition has been previously reported in non-sarcopenic patients [21], this study is, to the best of our knowledge, the first to show an association between deprescription and improved nutritional intake in sarcopenic patients with polypharmacy. Since the mainstay of treatment for sarcopenia includes nutritional therapy, this finding may be clinically important, suggesting that deprescribing and modifying polypharmacy in patients with sarcopenia may promote improvement in nutritional status. On the other hand, for each specific drug, increased number of PIMs [33] and anticholinergic load [34] are associated with malnutrition in older adults undergoing rehabilitation. However, the current study did not address the details of the specific medications that were reduced. In the future, high-quality prospective studies on the effects of specific medication reductions and dosages on nutritional status are required to fill the evidence gap.

Deprescribing was not statistically significantly associated with gains in muscle strength or mass in this study. This is possibly due to the study design, sample size calculation, and selection of confounders used for adjustment in the multivariate analysis, with the main outcome set as nutrient intake in this study. Therefore, the results of this study may not indicate that deprescribing is not effective in improving sarcopenia in these patients. However, since our findings showed that deprescribing may enhance the improvement in nutritional intake, it is possible that combining pharmacotherapy with exercise and nutritional therapy can further improve sarcopenia. Therefore, high-quality prospective studies are needed to examine the effects of deprescribing on improved muscle strength and muscle mass in sarcopenic patients with polypharmacy.

Nutrition, exercise, and pharmacotherapy from the perspectives of “rehabilitation pharmacotherapy” [35] and “rehabilitation nutrition” [36] are important in older adults undergoing rehabilitation. Sarcopenia is commonly found in about 50% of population undergoing rehabilitation [3] and is associated with outcomes such as ADL, dysphagia, and home discharge [5]. Therefore, establishing evidence regarding interventions for sarcopenia in patients undergoing rehabilitation is not only clinically important but also an urgent issue from a health economic perspective. However, at present, there is a lack of evidence on the efficacy of pharmacotherapy for sarcopenia. The current study suggested that drug management focusing on deprescribing in sarcopenic patients with polypharmacy enhances nutritional intake. Therefore, a multidisciplinary implementation of nutrition and exercise therapies, as well as medication management to modify polypharmacy, may further promote improvement in sarcopenia.

There were some limitations to this study. Firstly, this was a retrospective cohort study conducted in a single local hospital in Japan, which may limit the applicability for generalization. Secondly, the sample size was small, and we did not adjust for sufficient confounders in the multiple regression analysis. Finally, the effects of each specific drug were not taken into account. While some medications have side effects such as symptoms related to malnutrition and muscle damage, others increase motivation and appetite. In the future, it is needed to examine the impact of specific individual drug prescriptions on outcomes.

In conclusion, deprescribing was associated with improved nutritional intake in older sarcopenic patients with polypharmacy undergoing rehabilitation after stroke. Pharmacotherapy (combined with nutrition and exercise therapy) with a focus on modifying polypharmacy is important for the treatment of sarcopenia in these patients.

## Figures and Tables

**Table 1:** Baseline characteristics of participants.

	Total (*n =* 91)	Deprescribing Group (*n =* 39)	Non-Deprescribing Group (*n =* 52)	*p* Value
Age, y, mean (SD)	81.0 (7.5)	80.8 (8.2)	81.2 (7.1)	0.823 *
Sex, male, *n* (%)	44 (48.4)	15 (38.5)	29 (55.8)	0.102 ***
Stroke type				
Cerebral infarction, *n* (%)	61 (67)	23 (59.0)	38 (73.1)	0.157 ***
Cerebral hemorrhage, *n* (%)	24 (26.4)	14 (35.9)	10 (19.2)	0.074 ***
Subarachnoid hemorrhage, *n* (%)	5 (5.5)	2 (5.1)	3 (5.8)	1.0 ***
Stroke history, *n* (%)	37 (40.7)	17 (43.6)	20 (38.5)	0.622 ***
Premorbid mRS, median (IQR)	2 (0–3)	2 (0–4)	1.5 (0–3)	0.191 **
Onset-admission days, median (IQR)	15 (10–25)	17 (12–22)	15 (9–27)	0.779 **
Paralysis, *n* (%)				
Right/Left/Both	40 (44.0)/39 (42.9)/4 (4.4)	19 (48.7)/16 (41.0)/2 (5.1)	21 (40.4)/23 (44.2)/2 (3.8)	0.541 **
BRS, median (IQR)				
Upper limb/Hand-finger/Lower limb	4 (2–6)/5 (2–6)/5 (2–6)	4 (2–6)/4 (2–5)/4 (2–5)	4 (2–6)/5 (2–6)/5 (2–6)	0.619 **
FIM, score, median (IQR)				
- Total	36 (25–67)	33 (22–63)	43 (27–68)	0.227 **
- Motor	22 (14–51)	20 (13–40)	24 (16–52)	0.197 **
- Cognitive	15 (8–23)	14 (8–22)	15 (10–24)	0.472 **
FILS, score, median (IQR)	7 (2–8)	7 (2–7)	7 (6–9)	0.104 **
CCI, score, median (IQR)	3 (2–4)	3 (2–4)	3 (1–5)	0.766 **
Nutritional status, median (IQR)				
GNRI	91 (84–99)	91 (85–102)	90 (82–99)	0.767 **
BMI, kg/m^2^	21.3 (19.2–23.0)	21.4 (19.1–23.4)	20.7 (19.2–22.6)	0.411 **
Energy intake, kcal/kg/day	28.0 (24.1–33.3)	27.0 (23.7–31.6)	28.7 (24.6–34.0)	0.186 **
Protein intake, g/kg/day	1.1 (0.9–1.2)	1.0 (0.9–1.2)	1.1 (1.0–1.2)	0.307 **
Muscle-related variables, median (IQR)				
HG, kg				
Male	17.5 (10.2–22.0)	19.9 (6.0–22.4)	16.9 (11.6–21.7)	0.941 **
Female	9.2 (4.0–13.3)	9.3 (3.7–13.5)	9.2 (4.0–13.0)	0.571 **
SMI, kg/m^2^				
Male	6.1 (5.7–6.5)	6.0 (5.7–6.4)	6.1 (5.6–6.5)	0.766 **
Female	4.8 (4.0–5.1)	4.8 (4.1–5.2)	4.5 (4.0–5.1)	0.602 **
Laboratory data, mean (SD)				
Albumin, g/dL	3.4 (0.6)	3.4 (0.6)	3.4 (0.6)	0.707 *
C-reactive protein, g/dL	1.5 (2.6)	1.2 (2.5)	1.7 (2.7)	0.418 *
Hemoglobin, mg/dL	12.8 (1.8)	12.7 (2.1)	12.9 (1.7)	0.710 *
Length of stay, days, median (IQR)	107 (65–142)	102 (62–144)	113 (66–140)	0.904 **
Number of total medications, median (IQR)	8 (6–9)	9 (7–11)	7 (6–9)	0.003 **
Number of any PIMs, median (IQR)	1 (1–2)	1 (1–2)	1 (1–2)	0.365 **

* *t*-test; ** Mann–Whitney U test; *** chi-square test. BMI, body mass index; BRS, Brunnstrom Recovery Stage; CCI, Charlson’s Comorbidity Index; FILS, Food Intake Level Scale; FIM, Functional Independence Measure; GNRI, geriatric nutritional risk index; HG, handgrip strength; IQR, interquartile Range; mRS, modified Rankin Scale; PIMs, potentially inappropriate medications; SD, standard deviation; SMI, skeletal muscle mass index.

**Table 2:** Univariate analyses of outcomes between deprescribing and non-deprescribing group.

	Total (*n =* 91)	Deprescribing Group (*n =* 39)	Non-Deprescribing Group (*n =* 52)	*p* Value
Energy intake at discharge, kcal/kg/day, median (IQR)	29.1 (26.4–34.7)	30.5 (26.1–35.5)	28.6 (26.5–33.5)	0.227
Protein intake at discharge, g/kg/day, median (IQR)	1.2 (1.0–1.3)	1.2 (1.0–1.4)	1.2 (1.0–1.3)	0.912
HG at discharge, kg, median (IQR)				
Male	19.5 (14.4–25.1)	19.3 (15.5–24.1)	19.6 (14.2–25.4)	0.970
Female	11 (5.9–22.4)	12.3 (6.1–14.6)	9.7 (0.0–14.1)	0.493
SMI at discharge, kg/m^2^, median (IQR)				
Male	6.2 (5.7–6.8)	6.0 (5.7–6.3)	6.4 (5.5–7.0)	0.248
Female	4.9 (4.6–5.2)	4.8 (4.6–5.4)	4.9 (4.5–5.2)	0.985

HG, handgrip strength; IQR, interquartile Range; SMI, skeletal muscle mass index; SD, standard deviation.

**Table 3:** Multivariate linear regression analyses of outcomes at hospital discharge among older inpatients with sarcopenia and polypharmacy after stroke.

	Energy Intake at Discharge		Protein Intake at Discharge		HG at Discharge		SMI at Discharge	
	β	*p* Value	β	*p* Value	β	*p* Value	β	*p* Value
Age	−0.009	0.921	−0.170	0.141	−0.040	0.504	−0.066	0.359
Sex (male)	−0.092	0.331	−0.106	0.373	0.152	0.033	0.179	0.108
FIM-Total on admission	0.217	0.028	0.360	0.009	−0.068	0.368	−0.001	0.984
GNRI on admission	−0.538	<0.001	−0.726	<0.001	−0.082	0.215	0.192	0.026
Energy intake on admission	0.071	0.461	-	-	-	-	-	-
Protein intake on admission	-	-	0.151	0.208	-	-	-	-
HG on admission	-	-	-	-	0.827	<0.001	-	-
SMI on admission	-	-	-	-	-	-	0.586	<0.001
Change in number of drugs	−0.237	0.009	−0.242	0.047	−0.018	0.768	0.083	0.265

FIM, Functional Independence Measure; GNRI, geriatric nutritional risk index; HG, handgrip strength; SMI, skeletal muscle mass index.

## Data Availability

The data are not publicly available owing to opt out restrictions. Data sharing is not applicable.
